# Medical implementation practice and its medical performance evaluation of a giant makeshift hospital during the COVID-19 pandemic: An innovative model response to a public health emergency in Shanghai, China

**DOI:** 10.3389/fpubh.2022.1019073

**Published:** 2023-01-06

**Authors:** Minjie Chen, Yiling Fan, Qingrong Xu, Hua Huang, Xinyi Zheng, Dongdong Xiao, Weilin Fang, Jun Qin, Junhua Zheng, Enhong Dong

**Affiliations:** ^1^Department of Outpatient and Emergency Management, Renji Hospital, School of Medicine in Shanghai Jiao Tong University, Shanghai, China; ^2^Department of Neurosurgery, Renji Hospital, School of Medicine in Shanghai Jiao Tong University, Shanghai, China; ^3^Department of Orthopaedics, Renji Hospital, School of Medicine in Shanghai Jiao Tong University, Shanghai, China; ^4^Department of Administration, Renji Hospital, School of Medicine in Shanghai Jiao Tong University, Shanghai, China; ^5^Department of Pharmacy, Huashan Hospital, Fudan University, Shanghai, China; ^6^Department of Urology, Renji Hospital, School of Medicine in Shanghai Jiao Tong University, Shanghai, China; ^7^Department of Gastroenterology, Renji Hospital, School of Medicine in Shanghai Jiao Tong University, Shanghai, China; ^8^School of Nursing and Health Management, Shanghai University of Medicine and Health Sciences, Shanghai, China; ^9^Institute of Healthy Yangtze River Delta, Shanghai Jiao Tong University, Shanghai, China

**Keywords:** giant makeshift hospital, performance, TAN Bayesian network, COVID-19 pandemic, China

## Abstract

**Introduction:**

In confronting the sudden COVID-19 epidemic, China and other countries have been under great pressure to block virus transmission and reduce fatalities. Converting large-scale public venues into makeshift hospitals is a popular response. This addresses the outbreak and can maintain smooth operation of a country or region's healthcare system during a pandemic. However, large makeshift hospitals, such as the Shanghai New International Expo Center (SNIEC) makeshift hospital, which was one of the largest makeshift hospitals in the world, face two major problems: Effective and precise transfer of patients and heterogeneity of the medical care teams.

**Methods:**

To solve these problems, this study presents the medical practices of the SNIEC makeshift hospital in Shanghai, China. The experiences include constructing two groups, developing a medical management protocol, implementing a multi-dimensional management mode to screen patients, transferring them effectively, and achieving homogeneous quality of medical care. To evaluate the medical practice performance of the SNIEC makeshift hospital, 41,941 infected patients were retrospectively reviewed from March 31 to May 23, 2022. Multivariate logistic regression method and a tree-augmented naive (TAN) Bayesian network mode were used.

**Results:**

We identified that the three most important variables were chronic disease, age, and type of cabin, with importance values of 0.63, 0.15, and 0.11, respectively. The constructed TAN Bayesian network model had good predictive values; the overall correct rates of the model-training dataset partition and test dataset partition were 99.19 and 99.05%, respectively, and the respective values for the area under the receiver operating characteristic curve were 0.939 and 0.957.

**Conclusion:**

The medical practice in the SNIEC makeshift hospital was implemented well, had good medical care performance, and could be copied worldwide as a practical intervention to fight the epidemic in China and other developing countries.

## 1. Introduction

Since 2020, Shanghai has been experiencing a COVID-19 outbreak in 2 years. According to the Shanghai Municipal Health Commission, from the beginning of outbreak to May 2022, more than 590,000 cases have been identified, including 538,450 asymptomatic carriers (1). The rapidly increasing number of COVID-19 cases puts healthcare systems under extraordinary stress; epidemiological studies have shown that COVID-19 has a high rate of intrafamily transmission in China (2, 3), and it is especially difficult to monitor disease progress in the community (4, 5).

### 1.1. Construction of the makeshift hospitals and response to COVID-19 pandemic

Converting large-scale public venues into makeshift hospitals (MHs) is a popular means of rapidly addressing the coronavirus disease 2019 (COVID-19) outbreak and maintaining the smooth operation of a country or region's healthcare system during a pandemic (6). An MH (mobile cabin hospital or fangcang shelter hospital) is a modular health setup that provides multiple functions, including isolation, triage, basic medical care, frequent monitoring, rapid referral, and essential living needs (3). The successful employment of MHs in tackling the COVID-19 epidemic in Wuhan has been acknowledged worldwide (7, 8). The USA, the UK, and other countries have adopted a similar approach (9–11). The largest MHs in the world include Madrid's Ifema Emergency Field Hospital (5,500 beds, the largest in Europe) (12), the Wuhan Optics Valley Rihai MH (3,690 beds, the largest in China), and the Javits New York Medical Station (512 beds, the largest in the USA). China has built 120 MHs in Shanghai, including four giant hospitals (here we set > 10,000 beds as the capacity criterion for a giant MH), to ensure the swift quarantine and treatment of infected people and break the transmission path of COVID-19. Giant MHs can accommodate more infected patients for isolation treatment, and they enable limited medical resources to be centralized and integrated; thus, they play an important role in an epidemic.

Giant MHs also have shortcomings, including a lack of standard hospital medical facilities and that the integrated staff consists of medical professionals from different hospitals among the Chinese provinces. Moreover, a large number of patients are managed by temporarily recruited medical teams using an operative response mechanism catered to the emergency situation. Therefore, giant MHs pose great challenges to the Chinese healthcare system, medical treatment quality, and patient management.

**Figure 1:**
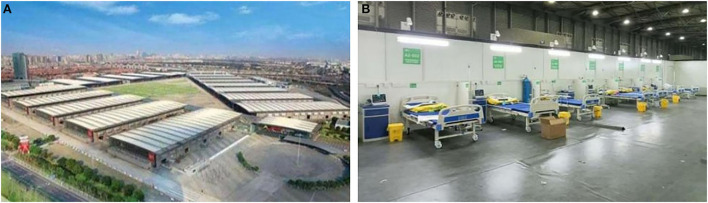
Exterior birdview **(A)** and interior view in subordinate cabin **(B)** of the SNIEC makeshift hospital.

Among the giant MHs, the Shanghai New International Expo Center (SNIEC) MH is the first >10,000-bed capacity MH in the world. It has a total construction area of over 300,000 square meters and 14,054 open beds; it was completed by 6,000 builders in 184 h. There are 10 ward cabins in the N (N1–N5) and W (W1–W5) medical areas. Figure 1A shows an exterior view of the SNIEC MH. During the whole operation stage, from March 31 to June 15, 2022, a total of 47,920 patients have been treated, setting a record of treating more than 14,000 infected patients in 1 day. From April 5, 2022, Renji Hospital affiliated to Shanghai Jiaotong University School of Medicine has been assigned to manage the SNIEC MH. Due to the sudden spike in the number of cases and limited resources designated to hospitals, the SNIEC MH was upgraded to two ward cabins: W1 and W2. Figure 1B shows an interior view of the SNIEC MH. The upgraded W1 and W2 ward cabins are equipped with 1,920 beds, with examination equipment (mobile CTs, ultrasound diagnostic devices, chest X-ray machines) and life support equipment (high-flow oxygen therapy device, ventilator, continuous renal replacement therapy, automated external defibrillator).

Like other large MHs, the SNIEC MH faces two major problems.

### 1.2. Problem 1: How can high-risk patients be identified and transferred to designated hospitals in a timely and effective manner?

COVID-19 is a highly contagious respiratory disease with varying symptoms. According to the eighth edition of the COVID-19 Prevention and Control Plan, COVID-19 cases can be divided into mild, moderate, severe, and critical types (13). However, the symptoms at onset are relatively mild, and a significant proportion of patients do not display symptoms prior to the development of respiratory failure. Clinically, this makes it difficult to predict the progression of disease severity in patients until respiratory failure occurs. The aging rate of Shanghai's population is the highest among megacities in mainland China and internationally. Limited medical facilities make it difficult to allocate advanced life support and primary disease treatment to critically ill patients over extended periods. At the peak of the epidemic, the SNIEC MH had eight ordinary cabins, two subordinate cabins, and more than 10,000 hospitalized patients. The ordinary cabin is referred to a simple basic dwelling in the makeshift hospital, which only receives asymptomatic patients or ones with symptoms of mild severity. Compared with the ordinary cabin, the subordinate cabin (also called sub-designated-hospital-cabin) is the transitional cabin between ordinary cabins and designated hospitals, and is built to receive and treat patients with symptoms of moderate severity. It also has additional functions such as imaging examination, facilitating the treatment of the common and underlying diseases for COVID-19 patients, and alleviating the rokecting demands for beds in designated hospitals. Due to the medical limitations of ordinary makeshift cabins, the theoretical indications for admission were asymptomatic and mildly severe disease. However, in practice, patients were usually admitted in batches; a community-based administrative unit organized transfers, resulting in great differences between the admission criteria and the patients' confirmed conditions. At peak times, thousands of patients per day were admitted in batches to hospitals. Therefore, the challenge arose as to how the SNIEC MH can timely and effectively screen for high-risk patients and refer them to designated hospitals for further medical treatment.

### 1.3. Problem 2: How can a temporally organized team achieve a homogeneous quality of medical care?

In contrast to traditional medical institutions and small- or medium-sized MHs, a medical team in the giant SNIEC MH includes more than 4,300 medical staff from more than 300 medical institutions among the Tianjin, Hubei, Jiangxi, Shanxi, Henan, Guizhou, and Shanxi provinces. This causes variations in the understanding of patients' conditions among the medical staff. Wearing personal protection equipment can impair the work efficiency of the medical staff. In addition, limited working hours and frequent handovers may cause deviations among the medical staff's familiarity with patients' conditions and full knowledge of the relevant medical procedures.

Existing literature on makeshift hospitals mainly focused on clinical characteristics (14–16), symptom dynamics, treatment strategies (17–19), and psychological distress (20–22) of COVID-19 patients in them. Studies illuminating medical care experiences of the MHs, especially a giant MH, and evaluating the medical care performance with quantitative methods were limited. Understanding the medical implementation practice and its performance evaluation of a giant makeshift hospital during the COVID-19 pandemic will assist in investigating the effectiveness of this novel centralized isolation approach in response to public health emergencies. Though unanimously building large-scale makeshift hospitals to cope with the COVID-19 is unlikely for all the countries worldwide, such possibility should not be excluded in the future. Due to the capricious trajectory of the COVID-19 pandemic and other potential emergencies accompanied by natural disasters, the healthcare system will be at the frontline of the emergency response team. Therefore, this study analyzed the medical implementation practices of the SNIEC giant MH in Shanghai and evaluated its medical practice performance using two methods. This will hopefully provide a practical reference for China and other countries to prevent and control similar events during public health emergencies.

Based on the aforementioned issues faced with the SNIEC MH in Shanghai, we put forward three research questions for the study: (1) how to identify and transfer COVID-19 patients to designated hospitals timely and effectively in the SNIEC MH? (2) how to ensure the homogeneous quality of medical care when the temporally organized medical team members from different regions cooperated together in the SNIEC MH? and (3) how to evaluate the operational performance of the aforementioned medical care practice in the SNIEC MH?

## 2. To solve problem 1: Construct two groups and develop a medical management protocol package to implement medical practice in the SNIEC MH

Two medical management organizational groups were constructed: an internal joint medical management group and an external comprehensive coordination group.

### 2.1. The joint medical management group

In order to achieve a homogeneous management quality, the SNIEC MH set up a joint medical office to be the managing department responsible for making plans, organizing, and controlling the medical activities in the entire hospital.

### 2.2. The external general coordination group

**Figure 2:**
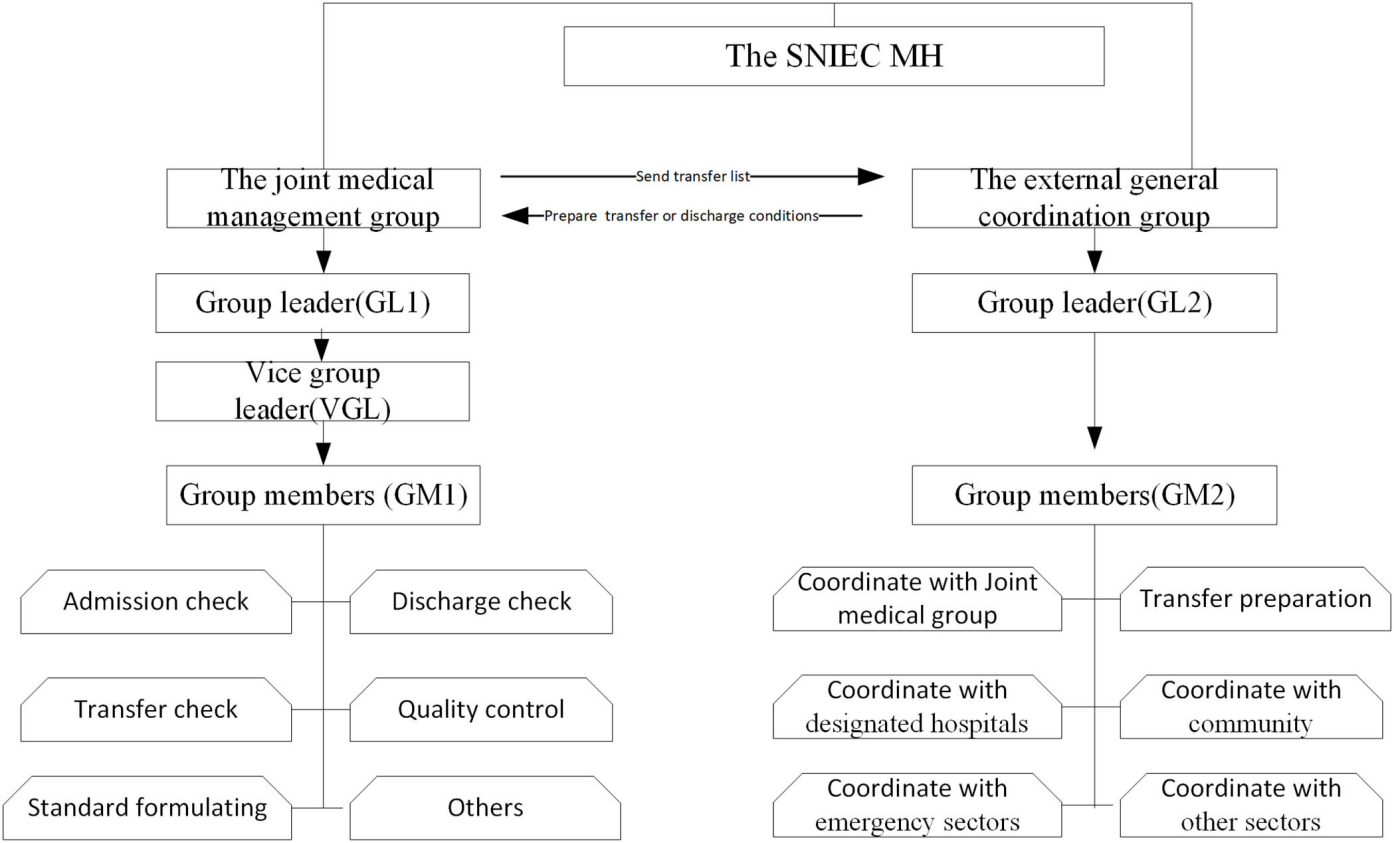
The work mechanism and organization structure of management groups in the SNIEC MH Group professional requirements: GL1: held by the chief physician and senior hospital administrator, VGL: held by the attending physician and the youth hospital manager, and GM1: consisting of some senior specialized senior chief physicians or attending physicians. GL2: held by one chief physician and one middle-aged hospital manager, GM2: consisting of hospital management majors, middle-aged hospital managers, attending physicians and junior medical technicians.

To ensure timely and effective communication regarding the discharge of recovered patients and referrals of essential patients, the SNIEC MH set up an external general coordination office. This group was connected with local government offices, health commissions, civil affairs bureaus, medical emergency centers, and major designated hospitals across 16 administrative districts. The external coordination team could coordinate with at least one designated hospital to reserve beds for potentially referred patients, establish a smooth referral system, and allow patients to be transferred quickly for medical emergencies. The work mechanism and organization structure of management groups in the SNIEC MH was displayed in Figure 2.

### 2.3. Developing a medical quality management package

**Figure 3:**
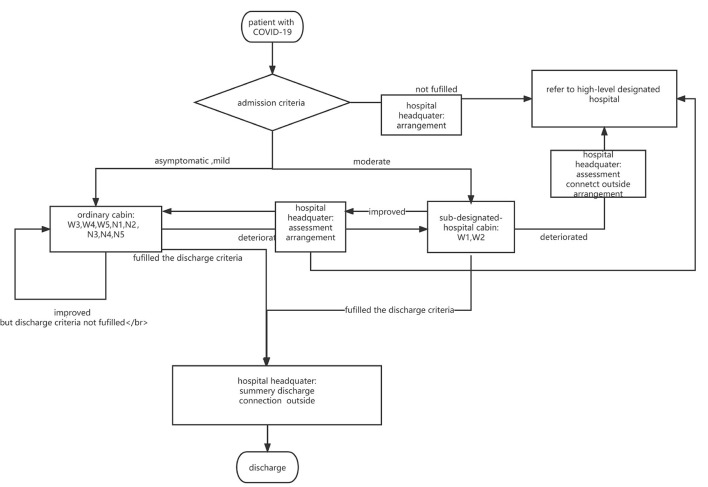
Procedure of patient screening and infow in the SNIEC makeshift hospital.

The SNIEC MH formulated a series of core management systems to ensure medical treatment quality, safety, and homogeneity of medical care. The core medical management systems included admission, referral, discharge, doctor rounds, consultation, assessing the conditions of elderly patients, nursing, duty and shift handover, case discussion, critical patient rescue, patient identity verification, medical record management, and management of antimicrobial drug classification. A flowchart of the patient screening and criteria for admission, discharge, and referral is shown in Figure 3.

#### 2.3.1. Admission criteria for ordinary cabins in the SNIEC MH

Generally, only asymptomatic patients and confirmed cases with mild severity could be accommodated (see the specific rules in Table S (A) of the Appendix file).

#### 2.3.2. Admission criteria of subordinate hospital cabin in the SNIEC MH

Generally, patients who are confirmed to have moderate severity can be accommodated (see specific rules in Table S (B) of the Appendix file).

#### 2.3.3. Discharge criteria in the SNIEC MH

See specific rules in Table S (C) of the Appendix file.

#### 2.3.4. Criteria for referral to a high-level designated hospitals

Due to the limited resources of designated hospitals, such as hospital beds and ventilators, clinicians were often faced with difficult decisions in which they must ration resources among patients. Therefore, criteria were formulated for referral to high-level designated hospitals. Generally, patients who are severe or critically ill will be referred (see specific rules in Table S (D) of the Appendix file).

## 3. To solve problem 2: Implement a multidimensional management mode to achieve homogeneous quality of medical care from a temporally organized team in the SNIEC MH

Internationally since 2020, many countries have implemented the integration of temporally-organized medical teams from various countries or regions to achieve the homogeneous quality of medical care to cope with COVID-19. For example, US has recruited volunteered health workers across the country to combat the outbreak of COVID-19. EU sent medical teams to Italy to help fight coronavirus. These medical workers from different regions were similar to the provincial supporting medical teams in our study. Although they did not know each other, and had different dialects, cultural background and medical practice features, they adopted various medical care modes, cooperated smoothly with each other to ensure the homogeneous quality of medical care.

Referring the aforementioned successful cases and experiences on integrated medical care teams of different origins in many countries, the SNIEC MH adopted a multi-dimensional management mode, as follows:

(A) Supervision of medical teams working 4-h shifts was conducted in each cabin. A shift leader was designated for each shift, and each patient was assigned to a clinician and the corresponding upper-level doctor.
(B) A multi-disciplinary cooperation rescue and emergency response team was formed consisting of senior professional physicians, in which medical experts were deployed to respond to emergencies at any time. The system was formulated so that the cabin was passed to a new team at 9 a.m. every day; medical experts discussed key patients at 11 a.m. each day to ensure the scientific nature of their medical treatment.
(C) A daily “observer” inspection system was established in which on-site screening of key patients, treatment guidance, coordinative examination, referral, and other medical affairs were conducted. The medical observer system can compensate for the early identification and diagnosis rate of critical patients and early treatment through daily inspection of key patients. Moreover, the medical observation system can overcome disadvantages in care heterogeneity (due to medical teams being from various provinces) and in the referral process by communicating with patients and their families. This improves the referral procedures and staff communication, which reduces the occurrence of medical disputes.

## 4. Evaluating the medical practice performance of the SNIEC MH using two methods

### 4.1. Data resource and variables

We retrospectively reviewed patients' data, which were randomly collected from the health information system (HIS) of the SNIEC MH, and eligible patients were selected if meeting the inclusion criteria as follows: 1 year and older; admitted up to May 23, 2022 (the final date of the data statistics); either discharged with recovery or referred to designated hospitals from March 31 to May 23, 2022. The patient outcomes were dichotomized into *discharge* (=0) or *referral* (=1) as the dependent variable. According to the existing literature (17, 23, 24), we considered some variables including demographic characteristics of the patients, such as age and sex, and their health-related profiles, including type of cabin, chronic disease, classified severity types, and length of stay as independent variables of interest in the study. Ethical approval was obtained through the University Human Research Ethics Committee (KY2022-102-B).

### 4.2. Analysis strategy

#### 4.2.1. Multivariate logistic regression method

All continuous variables were tested for normality. Means ± standard deviation were used for variables that fit a normal distribution; medians and interquartile ranges (IQRs) were used for variables with a skewed distribution. After univariate analysis identified significant contributors, a multivariate backward stepwise logistic regression method was used to predict the outcomes of patients.

#### 4.2.2. Tree-augmented naive Bayesian network method

Bayesian networks are suitable for multivariate analysis. A Bayesian network is a probabilistic graphical model for representing knowledge about an uncertain domain; each node corresponds to a random variable, and each edge represents the conditional probability for the corresponding random variables. A directed acyclic graph (DAG) is often used to represent complex variable relationships for a specific problem in a network structure, the directed edges of connected nodes represent causal relationships among variables. Conditional probability table (CPTs) are used to evaluate the correlation and intensity of variables (25). In recent years, Bayesian networks have been widely used for multivariate analyses in the medical field, including for analyzing clinical diagnoses and risk prediction (26–31). A tree-augmented naive (TAN) Bayesian network is a form of classic Bayesian network model that can deal with relevant variables and has good predictivity for multidimensional data (32). The TAN Bayesian network in this study uses a graphical diagram to provide a comprehensive way of representing relationships and influencing paths among variables of interest. The receiver operating characteristic curve (ROC) and area under the ROC curve (AUC) were used to evaluate the validity of the model.

All statistical analyses were performed using the SPSS version 23.0 statistical package (IBM Corp., Armonk, NY, USA). IBM SPSS Modeler 18.0 was used to build the TAN Bayesian network model.

## 5. Evaluation results

### 5.1. Basic characteristics

There were 41,941 eligible patients selected from the SNIEC MH. Among them, 41,558 (99.1%) were discharged with recovery, and 383 (0.9%) were referred to designated hospitals for further treatment. During the whole operation period of the SNIEC MH, no medical accidents or mortality occurred in the SNIEC MH. The Kolmogorov-Smirnov method test results (both the values of Asymp. Sig. =0.000< 0.05 for age and length of stay in hospitals) showed that none of the continuous features exhibited a normal distribution; therefore, medians and IQRs are reported. The median age was 43 years (IQR: 24, range: 1–102). The median length of stay was 8 days (IQR: 5). Of the 41,941 patients, 23,628 (56.3%) were male and 18,313 (43.7%) were female (Table 1). There were 4,575 (10.9%) moderate cases, 8,116 (19.4%) mild cases, and 29,250 (69.7%) were asymptomatic; 35,597 (84.9%) patients had no chronic disease, and 6,344 (15.1%) reported having a chronic disease. We divided the patients into four age-based groups. The groups included 33,191 (79.1%) of age ≤56 years, 8,112 (19.3%) of age 57–72 years, 504 (1.2%) of age 73–81 years, and 134 (0.3%) of age ≥82 years (Table 1). The other details are listed in Table 1.

**Table 1:** Patient characteristics, univariate and multivariate logistic regression analysis of factors related to the outcome of COVID-19 patients in SNIEC makeshift hospital.

**Items**	**Patient characteristics**		**Discharge patients** **(*N* = 41,558)**	**Referral** **Patients** **(*N* = 383)**	**Univariate** ***Chi square***		**Multivariate logistic regression**	
	**Variables**	**Patients,** ***n*** **(%)**			*χ^2^*	***P***	***OR*** **(95%** ***CI*****)**	***P***
Outcome	Discharge	41,558(99.1)	41,558	0	/	/	/	/
	Referral	383(0.9)	0	383	/	/	/	/
Type of cabin					*χ^2^ =* 968.118	*P* < 0.001	
	Ordinary cabin	37,393(89.2)	37,240(99.6)	153(0.4)			1.000	
	Subordinate hospital Cabin	4,548(10.8)	4,318(94.9)	230(5.1)			0.041 (0.012–0.148)	< 0.001
Gender					*χ^2^ =* 39.557	*P* < 0.001		
	Male	23,628(56.3)	23,473(99.3)	155(0.7)			1.000	
	Female	18,313(43.7)	18,085(98.8)	228(1.2)			1.602 (1.267–2.026)	< 0.001
Age (years)					*χ^2^ =* 4,707.840	*P* < 0.001		
	1–56	33,191 (79.1)	33,028 (99.5)	163 (0.5)			1.000	
	57–72	8,112 (19.3)	8,010 (98.7)	102 (1.3)			0.687 (0.522–0.905)	0.007
	73–81	504 (1.2)	45 (8.9)	459 (91.1)			1.565 (1.043–2.350)	0.031
	Above 82	134 (0.3)	73 (54.5)	61 (45.5)			17.393 (10.550–28.676)	< 0.001
Classified for severity types					*χ^2^ =* 1,025.791	*P* < 0.001		
	Asymptomatic	29,250(69.7)	29,162 (99.7)	88 (0.3)			1.000	< 0.001
	Mild	8,116(19.4)	8,056(99.3)	60(0.7)			1.872 (1.331–2.641)	< 0.001
	Moderate	4,575(10.9)	4,340(94.9)	235(5.1)			100.528 (28.005–260.862)	< 0.001
Chronic disease or not					*χ^2^ =* 1,726.967	*P* < 0.001		
	No	35,597(84.9)	35,562(99.9)	35(0.1)			1.000	
	Yes	6,344(15.1)	5,996(94.5)	348(5.5)			36.364 (25.113–52.656)	<0.001
Length of hospital stay	/	/	/	/	/	/	0.728 (0.697–0.759)	<0.001

### 5.2. Results of univariate and multivariate logistic regression analysis

The characteristics of the discharged and referred patients were compared using univariate analysis (Table 2). The factors associated significantly with the outcome of patients were type of cabin (*P* < 0.001), sex (*P* < 0.001), age (*P* < 0.001), severity type (*P* < 0.001), and chronic disease (*P* < 0.001). Multivariate logistic regression analysis, using the backward stepwise method in the two groups of patients, showed that the risk factors associated significantly with referral were female sex (OR: 1.602, *P* < 0.001), age 73–81 years (OR: 1.565, *P* = 0.031), age ≥ 82 years (OR: 17.393, *P* < 0.001), mild disease (OR: 1.872, *P* < 0.001), moderate disease (OR: 100.528, *P* < 0.001), and chronic disease (OR: 36.364, *P* < 0.001). The protective factors for referral patients were age 56–72 years (OR: 0.687, *P* = 0.007), subordinate hospital cabin (OR: 0.041, *P* < 0.001), and length of hospital stay (OR: 0.728, *P* < 0.01) (Table 1).

**Table 2:** Predictor importance of Bayesian network.

**Variables**	**Predictor importance**
Having a chronic disease	0.63
Age	0.15
Type of cabin	0.11
Gender	0.05
Length of hospital stay	0.04
Classified severity types	0.02

### 5.3. Results of TAN Bayesian network method

**Figure 4:**
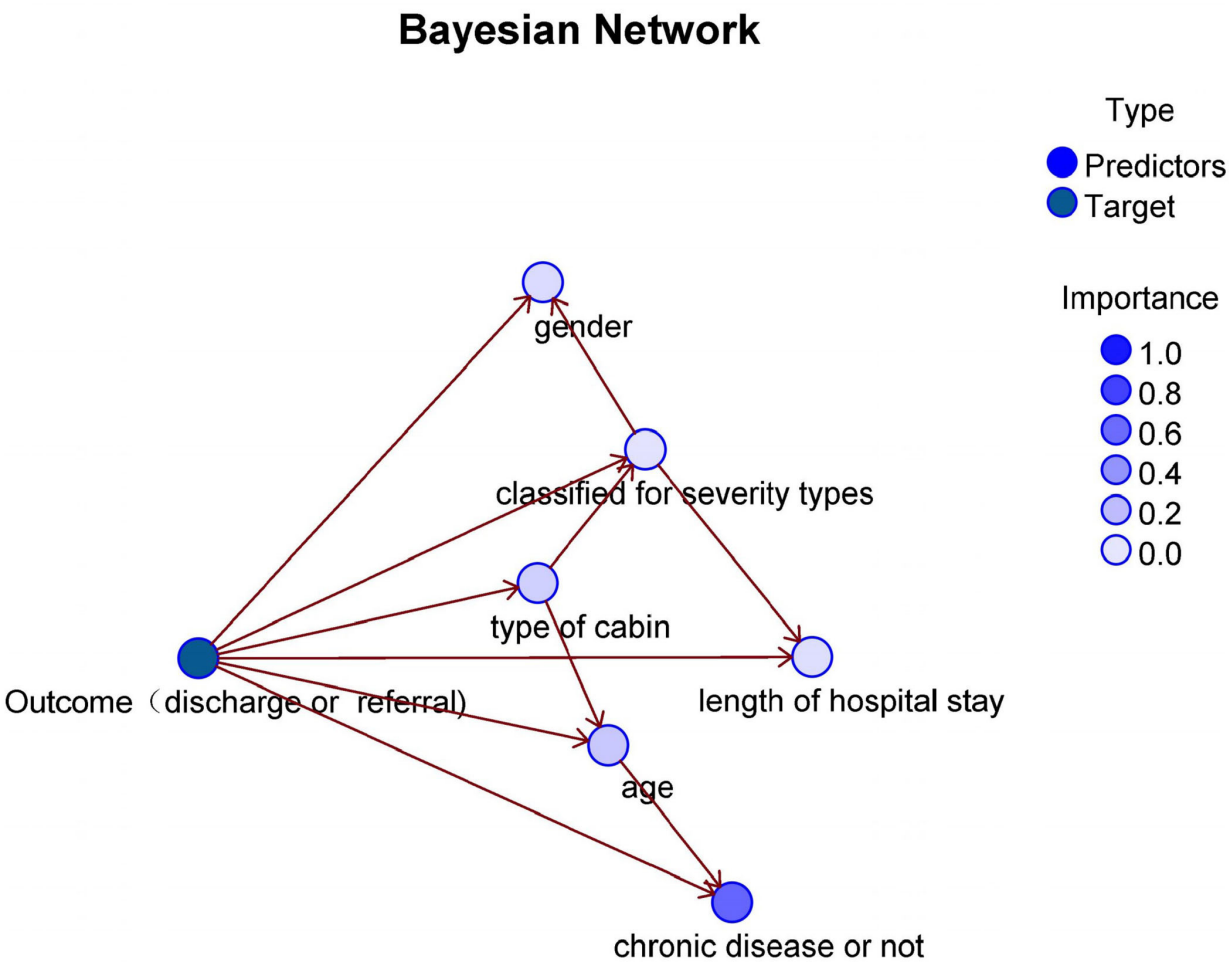
Directed acyclic graph of Bayesian network.

SPSS modeler 18.0 was used to establish a Bayesian network model of factors that influence patients' outcomes. We randomly divided the dataset into training and test sets at a 70:30 ratio (29,272 and 12,669 records, respectively). We set sex, age, type of cabin, presence of chronic disease, classification of severity type, and length of stay as import variables; the outcome (discharge or referral) was the target variable. DAGs and CPTs were mined using the TAN model of the Bayesian network. In a DAG, each node representing an occurring event represents a variable, and all nodes pointing to node X are called the parent nodes of X. A Bayesian network edge indicates the probability that the event of the child node will occur if the event of the parent node has occurred (the root node has no probability). Parent and child nodes must be directly related. The DAG is shown in Figure 4. In the DAG, the darkness of the input variable's color indicates the importance of the prediction of the target variable. The input variable with the darkest color is a chronic disease, the nodes in the DAG correspond to the variables in the model, and each node has a CPT (Figure 4). Figure 4 and Table 2 demonstrate the importance of these variables. We found that all six input variables had effects on the target variable; the most important variables were chronic disease, age, and type of cabin (0.63, 0.15, and 0.11, respectively). The TAN Bayesian network not only computes the probability distributions of the child nodes given the values of their parent nodes, but also the distribution of the parent nodes given the values of their children. That is, they can both proceed from causality and deduce the probabilities of different causes given the consequences. The conditional probability tables present all possible conditional probabilities when each node is conditioned on its parent node (Tables 3A–C).

Table 3Conditional probabilities of having a chronic disease or not, age and type of cabin.

**Table:** 

**(A) Having a chronic disease or not**			
**Parent nodes**		**Probability**	
**Age**	**Outcome**	**Chronic disease (No)**	**Chronic** **disease (Yes)**
1–56	Referral	0.15	0.85
57–72	Referral	0.11	0.89
73–81	Referral	0.04	0.96
No less than 82	Referral	0.05	0.95

**Table:** 

**(B) Age**					
**Parent nodes**			**Probability**		
**Type of cabin**			**Age**		
**Outcome**		**1–56**	**57–72**	**73–81**	**No less than 82**
Ordinary	Referral	0.67	0.26	0.06	0.01
Ordinary	Discharge	0.81	0.18	0.00	0.00
Subordinate	Referral	0.26	0.31	0.15	0.28
Subordinate	Discharge	0.63	0.30	0.07	0.01

**Table:** 

**(C) Type of cabin**		
**Parent nodes**	**Conditional probability**	
**Outcome**	**Ordinary cabin**	**Subordinate** **Hospital cabin**
Referral	0.42	0.58
Discharge	0.90	0.10

### 5.4. Model validation

**Figure 5:**
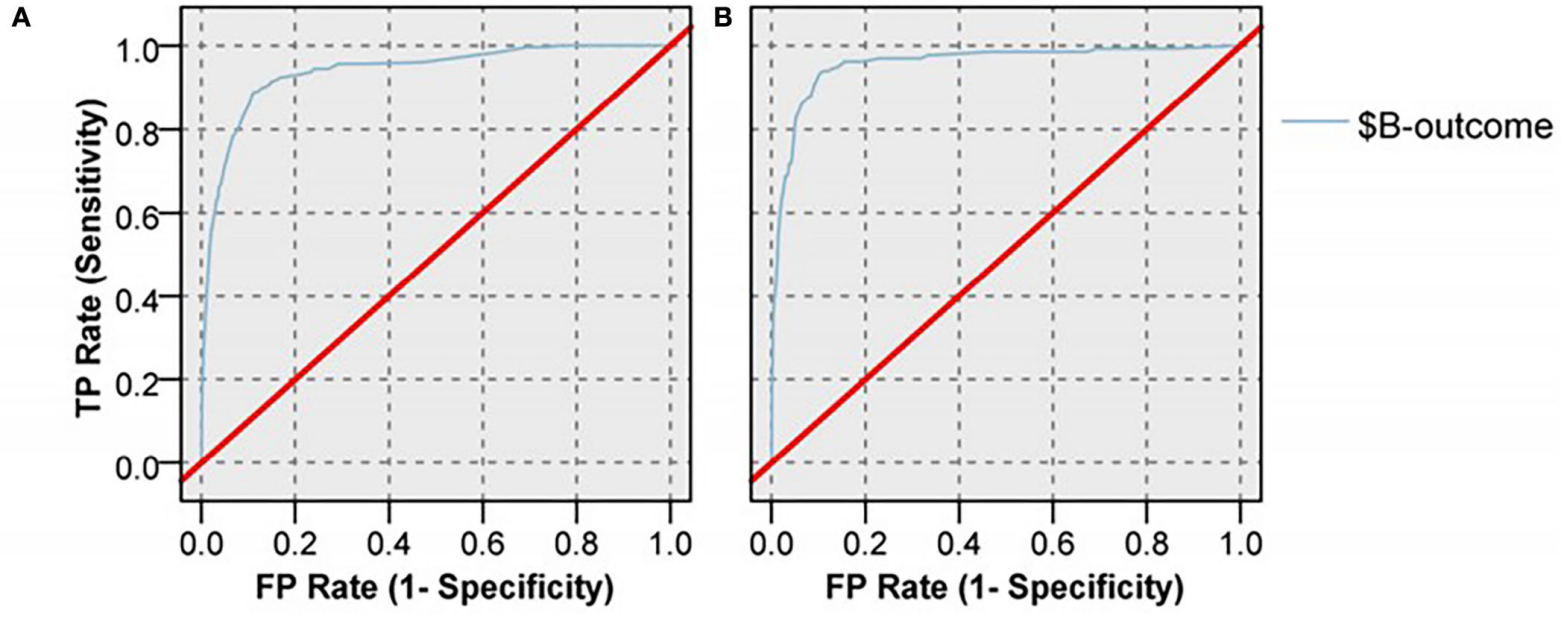
ROC curve of Bayesian network model in training dataset **(A)** and test dataset **(B)**.

The performance of the model was assessed using the AUC of the ROC curve. The ROC was plotted by obfuscation matrix. In the field of machine learning, the confusion matrix is a situation analysis table that summarizes the prediction results of the classification model. It is a visualization tool used to test the effects of the classifier. In the form of a matrix, the records in the dataset were predicted based on real classification values and classification models. The classification results were compared and summarized; each row of the matrix represents the real category, and each column represents the predicted category. The AUC represents the ROC curve and has a value between 0 and 1. The closer the ROC curve is to the upper left corner, the greater the AUC value is; this indicates that the greater the prediction capability, the better the model. The constructed Bayesian network model had a good predictive value (Figure 5, Table 4). The overall correct rates of the model-training dataset partition and test dataset partition were 99.19 and 99.05%, respectively; the respective AUC values were 0.939 and 0.957.

**Table 4:** The validation of TAN Bayesian network.

	**Training dataset**		**Test dataset**	
Correct	29,036	99.19%	12,549	99.05%
Wrong	236	0.81%	120	0.95%
AUC	0.939		0.957	

AUC, Area under the ROC curve.

## 6. Discussion

COVID-19 has upended myriad aspects of life, particularly the receipt of usual medical care. Routine medical observation and treatment might not be available during the pandemic, especially in developing countries, because medical interactions often violate social distancing protocols, and people in need of medical care are often at risk for adverse outcomes from COVID-19. To cope with and adapt to this situation, giant MHs are specially constructed to respond to COVID-19 and other public health emergencies. They need to be able to screen out COVID-19-infected patients at risk of deterioration and transfer them to high-level designated hospitals. However, it is also necessary to alleviate pressure on high-level designated hospitals for admission and treatment. Achieving this trade-off is a challenge for medical managers in MHs. Moreover, it is crucial to implement homogeneous cabin management among the different temporally organized medical teams in this large medical setting, especially for standardizing the criteria for admission, discharge, and transfer of patients.

In the SNIEC MH, we constructed two groups and developed a medical management protocol to conduct patient screening and treatment. A multi-dimensional management mode was designed to achieve homogeneous quality of medical care by a temporarily organized team. To evaluate the operational medical performance of the SNIEC MH, we constructed a TAN Bayesian network model to assess the outcome of COVID-19–infected patients and the model was validated. It was shown in the DAG that the *type of cabin* is the parent node of the *classified severity types*. This means that the patients in the SNIEC MH were screened for suitability of transfer to an ordinary cabin or a sub-designated–hospital cabin according to their severity type classification. Consequently, the results were consistent with the operational principals of the SNIEC MH, identifying that the admission standards for ordinary cabins and the sub-designated–hospital cabins were implemented well in practice. DAG showed that *length of stay* and age, and *type of cabin* and *chronic disease*, had no direct linear segments. This indicates that these contributors were independent of each other. Furthermore, the DAG identified that the parent node of the *length of stay* is *classified severity types*, indicating that the discharge standards formulated by the SNIEC were implemented appropriately.

Through the multivariate logistic regression model, our research showed that gender, age (73–81 years and ≥ 82 years), severity type (mild disease and moderate disease), and chronic disease were risk factors for the referral of COVID-19 patients, while age 56–72 years, and type of cabin (subordinate hospital cabin) were protective factors for referral. Furthermore, the predictive importance of the Bayesian network showed that having a chronic disease was the most important indicator for predicting the outcome of patients. The importance of influencing factors was 0.63; the importance of age was 0.15, which was higher than that of other factors. These findings were in concordance with some previous studies (23, 33–40). A retrospective study found that the patient's age, hypertension and heart disease are independent risk factors for the progression of COVID-19 patients in Fangcang shelter hospitals (33). A study found older age (over 65 years) was associated with higher odds of progression to severity of COVID-19 (34). Many studies have shown that COVID-19 patients with a history of chronic diseases were more difficult to cure, and the underlying chronic disease was also more likely to develop into a severe disease or even causing death (35–38). These studies have shown that for the elderly patients and patients with potential comorbidities, more attention, early referral or timely intervention are needed to avoid the development of severe illness or death. However, the association between gender and COVID-19 patients' outcomes is currently inconclusive in the field of COVID-19 research. In our study, female sex was a risk factor associated with referral of patients with COVID-19. This may be due to the distribution of patients' age with 70.9% females were in the age group of ≥ 82 years in the study. Conversely, some studies found male sex was associated with a worse outcome of patients with COVID-19 in an elderly hospitalized population (23, 39). Although the reasons for this gender difference are unknown, it has been suggested that males and females differ in their immunological responses and in their susceptibility to infection (40, 41).

This result confirms that the transfer criteria were implemented well-regarding team management at the SNIEC MH. During the entire operational stage of the SNIEC MH, there were zero deaths and zero medical accidents. It was shown that, in response to the aforementioned challenges of the SNIEC MH, the medical management quickly established an adaptable organizational system that allowed team members to dilute their previous medical roles in their original hospitals. This enabled them to smoothly adapt to the new organizational structure, give priority to teamwork efficiency, and work with the various medical rules and regulations to achieve homogeneous management of the medical staff in the SNIEC MH.

This study has several strengths that contribute to the existing literature. First, the study established a mechanism, verified by the TAN Bayesian network model, to predict referrals of patients that can assist doctors in making decisions and improve referral efficiency. To the best of our knowledge, this is the first study to focus on the medical practice of a giant MH and describe how it works in response to a public health emergency. The management mode has been successfully carried out, proven to be effective, and can be copied and utilized worldwide. Second, although we included only six characteristic variables, our model achieved a trade-off between the minimal number of features and the capacity for good prediction by avoiding overfitting. This finding shows that evaluation of a small number of patient characteristics, such as chronic disease or age, by the TAN Bayesian network model could help medical staff rapidly screen patients to determine whether they should be discharged or transferred, making full use of the current healthcare infrastructure in the event of public health emergencies. Third, this research contributes to the development of medical quality management tools for giant MHs, which will help clinicians conduct decision-making analyses and risk assessment during a pandemic or for other illnesses or injuries caused by large-scale public emergencies, including a mass poisoning or natural disaster.

### 6.1. Limitations

This study had some limitations. First, the study was conducted at a single center. Although the dataset contained more than 40,000 cases, a further prospective multicenter study using external validation methods could be needed to assess the medical care performance of the model for the generalizability of the study findings. Second, although most parameters were documented objectively, some independent variables were self-reported, such as a history of chronic diseases; this may have introduced a recall bias in the study.

## 7. Conclusion

A public health emergency in a megacity with a population over 10-million will likely see the number of patients spike swiftly during a pandemic outbreak. Therefore, it is important to construct giant MHs to accommodate infected cases and relieve the strain on regular healthcare services. MHs played an important role as an intermediate platform for COVID-19 patients. As the biggest MH in China, the SNIEC MH developed a medical management protocol package and a multi-dimensional temporally organized integrated team to screen patients and transfer them effectively and achieve homogeneous quality of medical care. This was confirmed by multivariate logistic regression analysis and the TAN Bayesian network method to be implemented well and have good performance.

## Data availability statement

The raw data supporting the conclusions of this article will be made available by the authors, without undue reservation.

## Ethics statement

The studies involving human participants were reviewed and approved by the Renji Hospital Affiliated to the Shanghai Jiaotong University School of Medicine's Institutional Review Board. Written informed consent was not required in accordance with the ethics approval.

## Author contributions

MC, JQ, JZ, and ED designed the study together, acquired the data, and developed the statistical plan, performed the statistical analysis, and interpreted the analysis. QX, YF, DX,WF, HH, and XZ carried out the survey. MC, JZ, and ED drafted and revised the manuscript. All authors read and approved the final manuscript.
