# Supplementary material for: Medical implementation practice and its medical performance evaluation of a giant makeshift hospital during the COVID-19 pandemic: An innovative model response to a public health emergency in Shanghai, China
Source: Front Public Health. 2023 Jan 6;10:1019073. doi: 10.3389/fpubh.2022.1019073 (PMC9853970; doi:10.3389/fpubh.2022.1019073)
Supplement: Supplementary file 1 [file Table_1.docx]

**Table S | The Specific rules of patient management in the SNIEC makeshift hospital**

| 1. **: Admission criteria of ordinary cabin in SNIEC makeshift hospital** | |
| --- | --- |
|  | 1.Patients with asymptomatic or mild severity;  2.Patients with ability take care of themselves independently；  3.Patients without basic diseases of respiratory system;  4.Patients without cardiovascular system and cerebrovascular system in acute stage;  5.Patients without severe organ dysfunction:such as requiring blood purification; advanced tumors, decompensated liver cirrhosis;  6.women without in pregnancy and perinatal period;  7.Patients without history of mental and psychological diseases. |
| **(B)：Admission criteria of sub-level-hospital cabin in SNIEC makeshift hospital** | |
|  | Details of criteria are as following:  1.Patients with moderate severity;  2.Patients with serious heart, liver, lung, kidney, brain and other basic diseases in stable stage, excluding patients requiring hemodialysis and radiotherapy and chemotherapy;  3. Patients in the acute stage who do not require surgical treatment and endoscopic intervention ;  4.Women in pregnancy, but not in high risk. |
| **(C): Discharge criteria in SNIEC makeshift hospital** | |
|  | 1. Afebrile for at least 3 days.  2. Respiratory symptoms have improved significantly.  3. Pulmonary inflammation has decreased markedly according to Radiology.  4. Ct values of N gene and ORF gene in two consecutive Covid-19 nucleic acid tests were ≥35, and the limit value was 40 by fluorescence quantitative PCR. The sampling interval was at least 24 hours.  Or negative test ofCovid-19 nucleic acid for two consecutive times, limit value of fluorescence quantitative PCR method below 35, sampling interval of at least 24 hours) |
| 1. **：Criteria for referral to high-level designated hospital** | |
|  | 1.Severe and critically ill patients with COVID-19;  2.Patients with serious heart, liver, lung, kidney, brain and other basic diseases, including hemodialysis, or acute aggravation of chronic organ dysfunction;  3.Acute emergency, such as acute coronary syndrome, acute pulmonary embolism, acute gastrointestinal bleeding patients;  4.High-risk pregnant women;  5.Patients without the ability to live independently;  6.Mental illness, manic patients;  7.Unattended children under 7 years of age;  8.children appear persistent high fever, shortness of breath, assisted breathing (nasal wing flapping, three depression signs), lethargy, convulsion, feeding or feeding difficulties, dehydration signs and other conditions;  9.Having various clinical conditions that are potentially life-threatening;  10.Other special emergency causes(to be noted). |
